# Supplementary material for: Genomic analysis of Ostreococcus tauri-infecting viruses reveals a hypervariable region associated with host–virus interactions
Source: Virus Evol. 2026 Feb 11;12(1):veaf096. doi: 10.1093/ve/veaf096 (PMC12906663; doi:10.1093/ve/veaf096)

a.

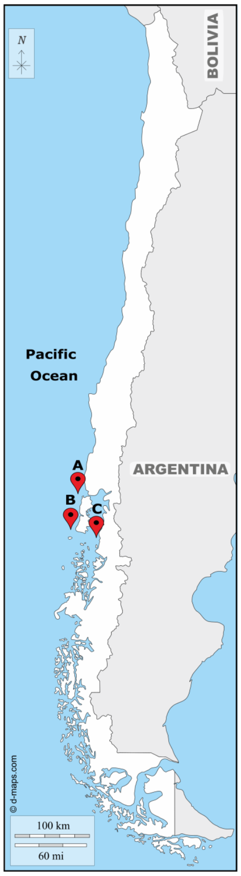

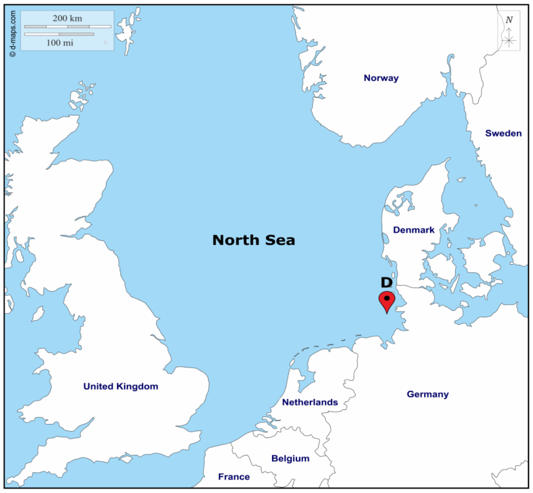

Sampling sites	Virus	Host strain
A	OtV19-O	*O. tauri* RCC4221
	OtV19-P	
B	OtV19-R	
C	OtV19-T1	
	OtV19-T2	
	McV20-T	*M. commoda* RCC827
D	OtV-Sylt2-5	*O. tauri* RCC4221

b.

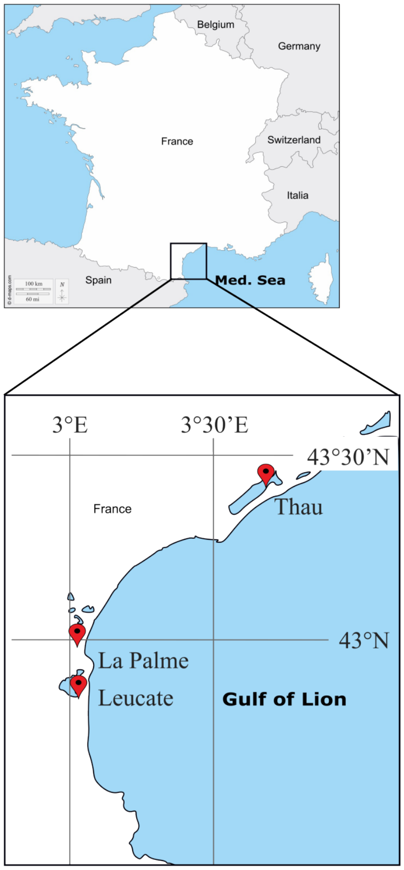

Supplement: supplementary-material [file supplementary-material.zip › FigureS1_veaf096.pdf]
