# Supplementary figures and images for: Genomic analysis of Ostreococcus tauri-infecting viruses reveals a hypervariable region associated with host–virus interactions
Source: Virus Evol. 2026 Feb 11;12(1):veaf096. doi: 10.1093/ve/veaf096 (PMC12906663; doi:10.1093/ve/veaf096)

a.

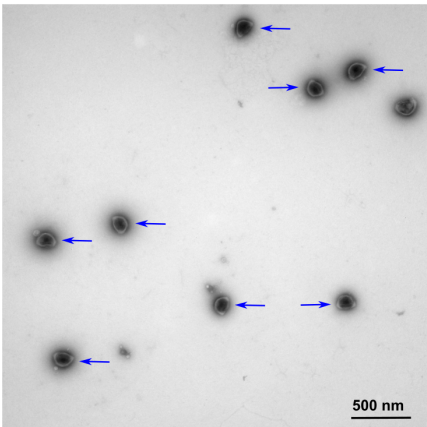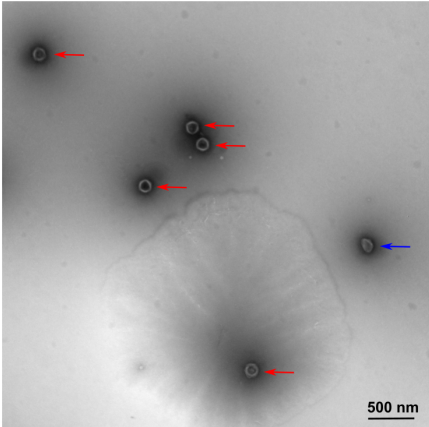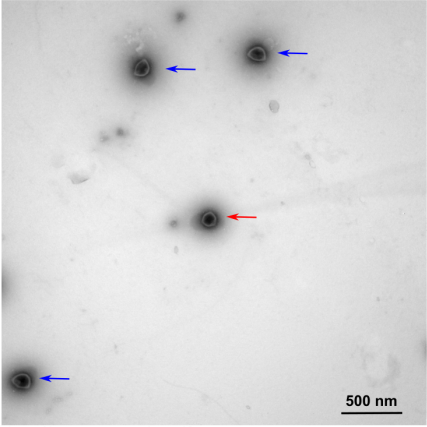

b.

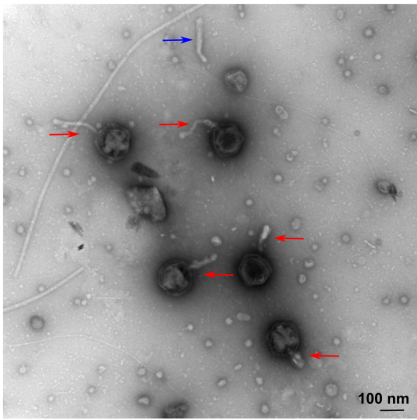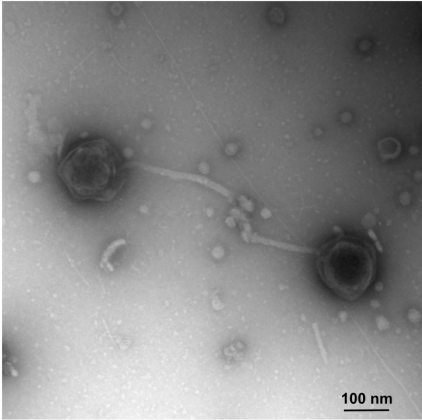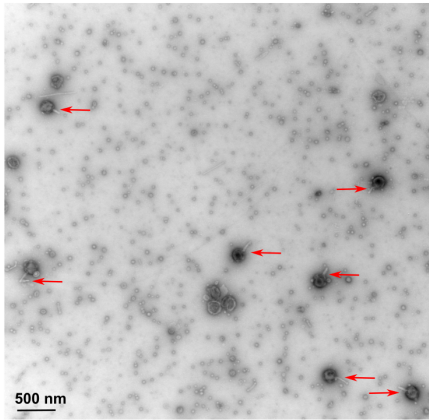

Supplement: supplementary-material [file supplementary-material.zip › FigureS10_veaf096.pdf]

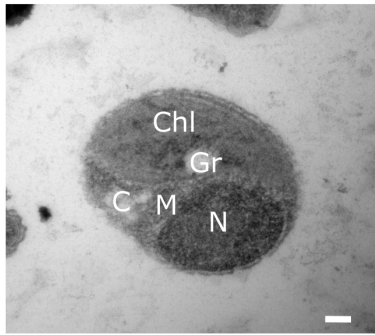

Uninfected

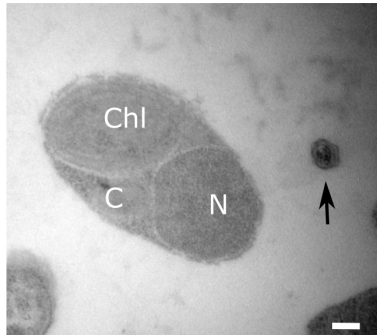

5 mpi

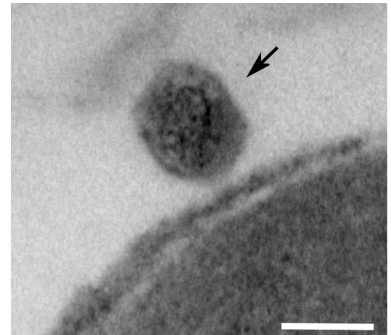

30 mpi

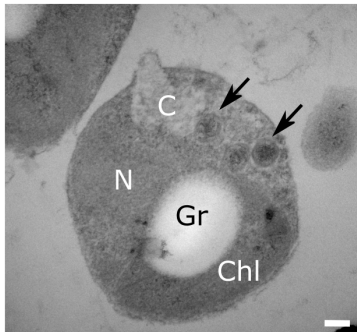

4 hpi

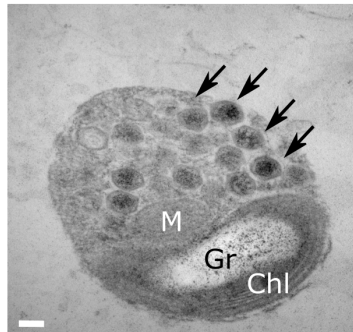

12 hpi

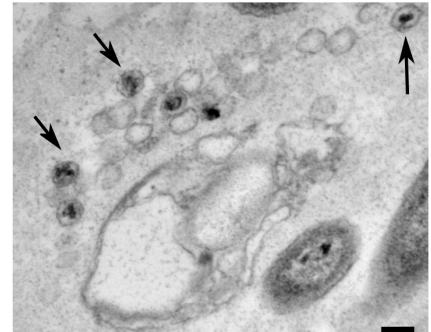

72 hpi

Supplement: supplementary-material [file supplementary-material.zip › FigureS11_veaf096.pdf]

OtV06-12-00065

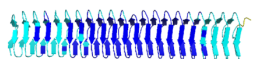

OtV09-565-00065

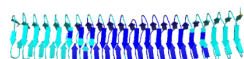

OtV19-O-00074

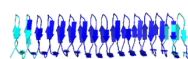

OtV09-582-00074

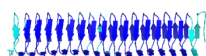

OtV5-070

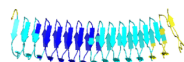

OtV09-582-00075

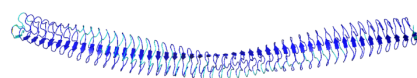

OtV5-071

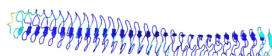

Supplement: supplementary-material [file supplementary-material.zip › FigureS12_veaf096.pdf]

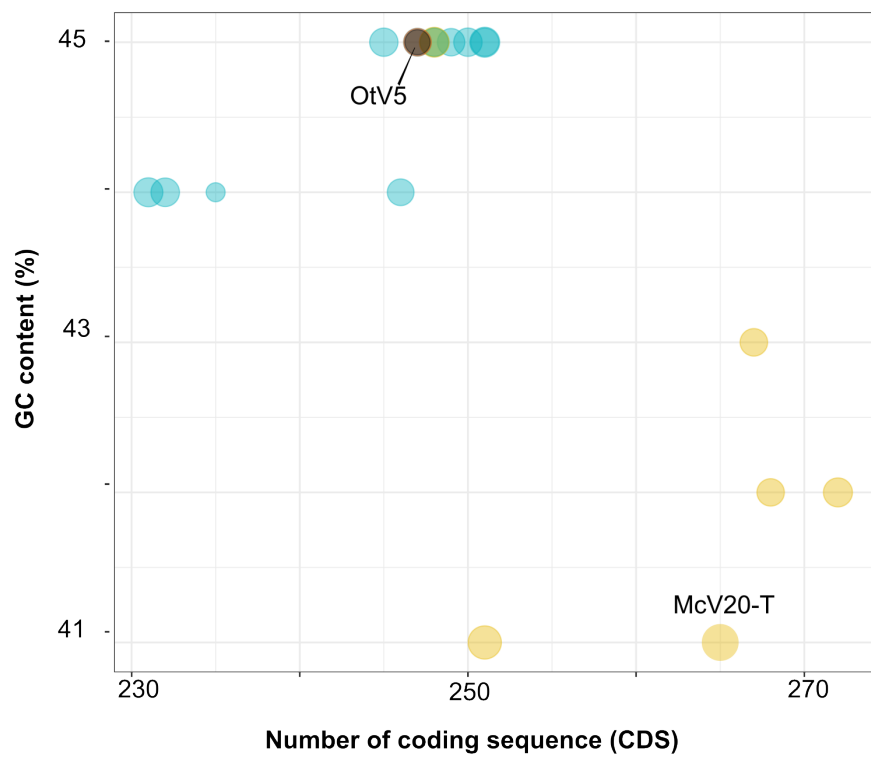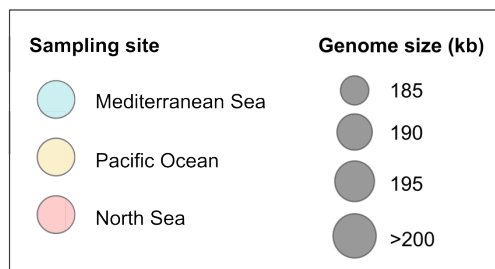

Supplement: supplementary-material [file supplementary-material.zip › FigureS2_veaf096.pdf]

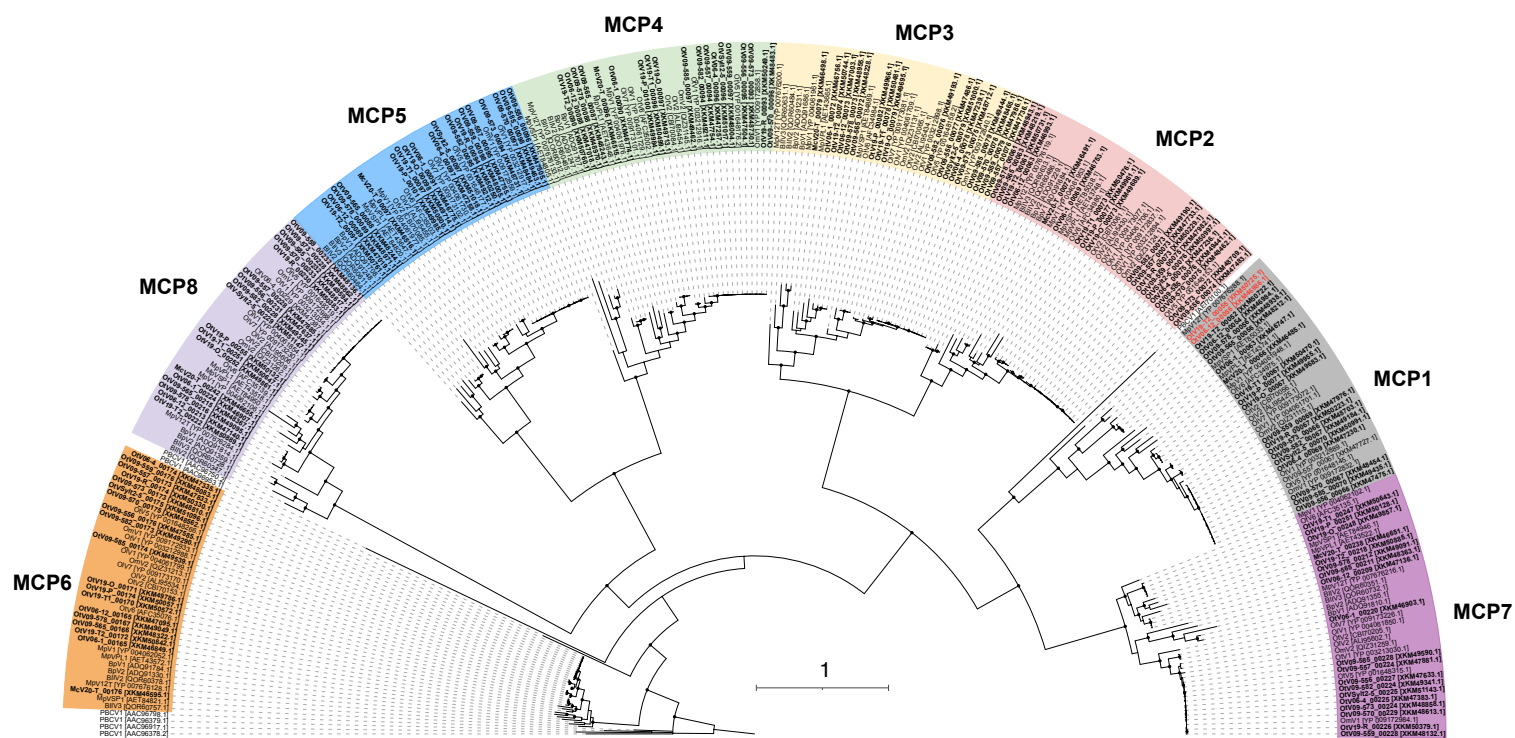

Supplement: supplementary-material [file supplementary-material.zip › FigureS3_veaf096.pdf]

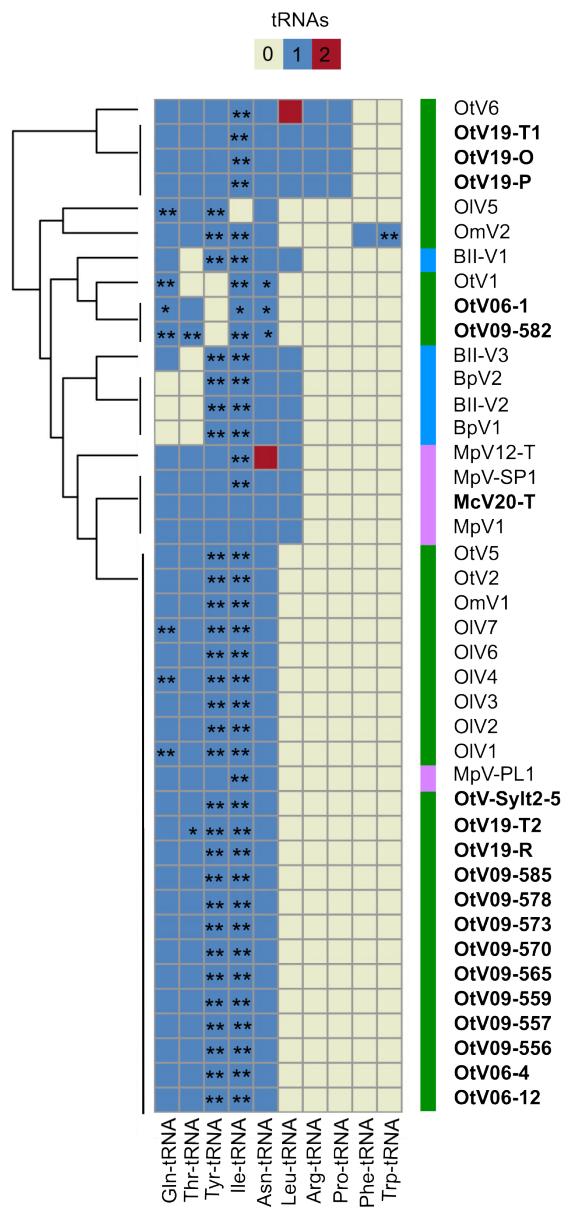

Supplement: supplementary-material [file supplementary-material.zip › FigureS4_veaf096.pdf]

a. Concatenated core proteins

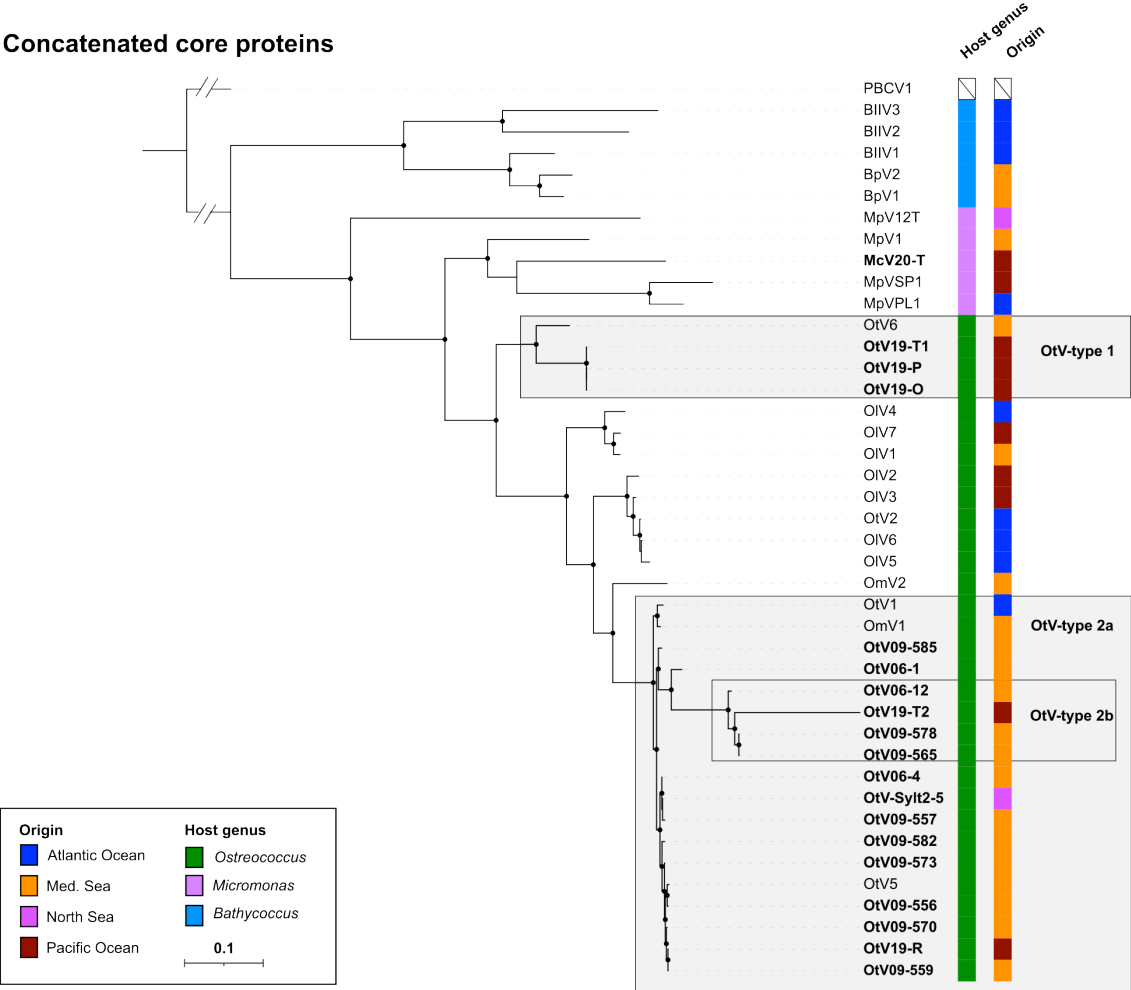

b. Single DNA polymerase B protein

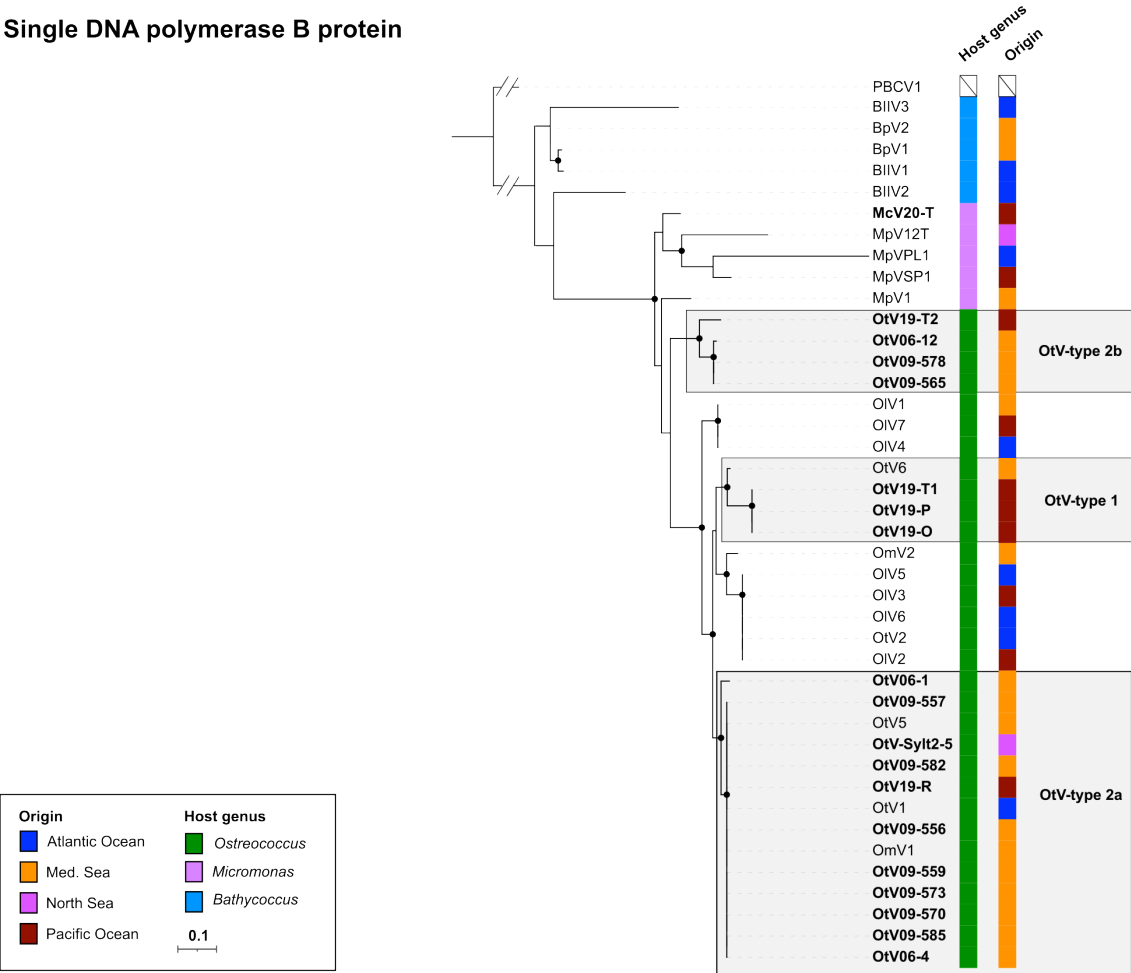

Supplement: supplementary-material [file supplementary-material.zip › FigureS5_veaf096.pdf]

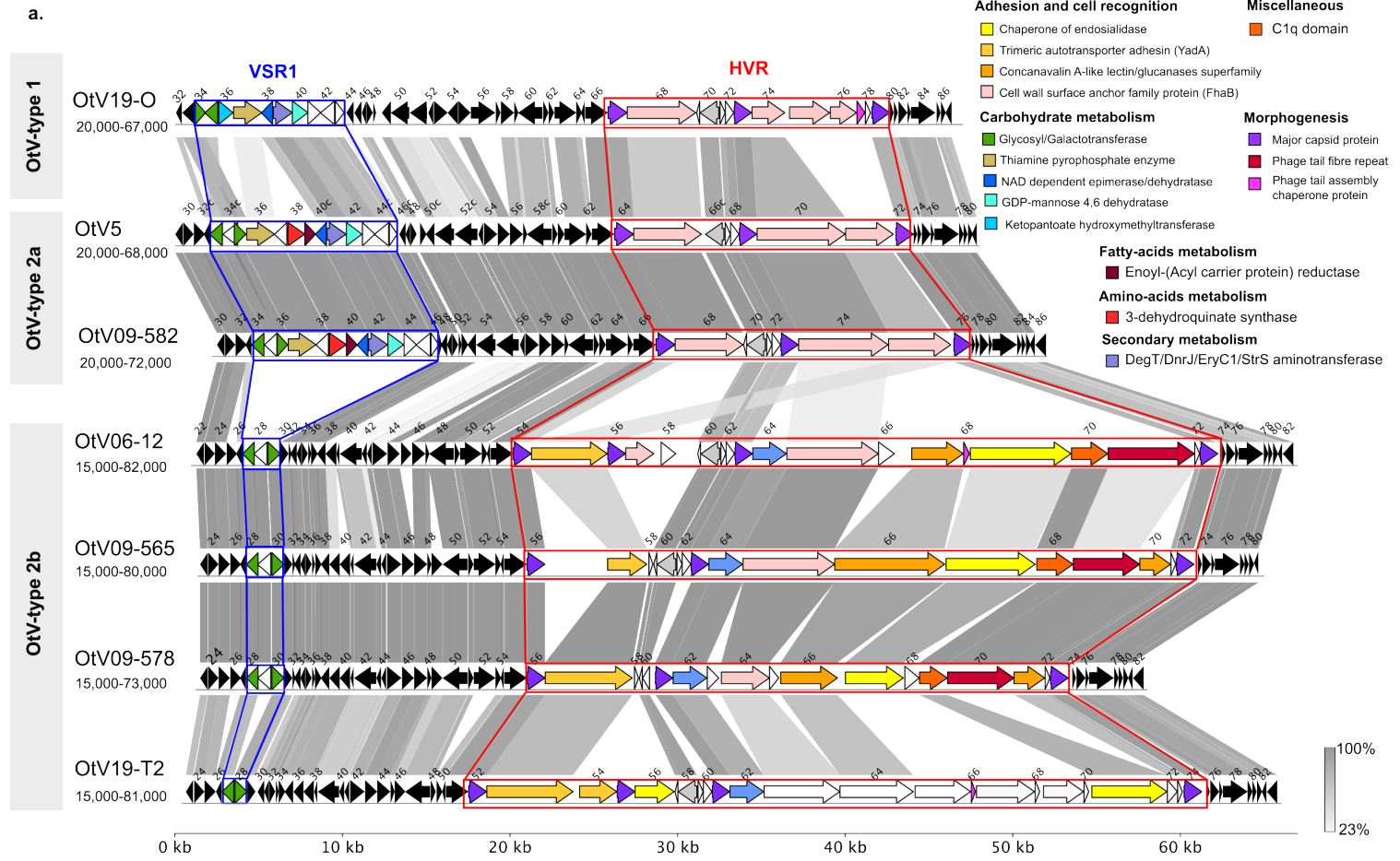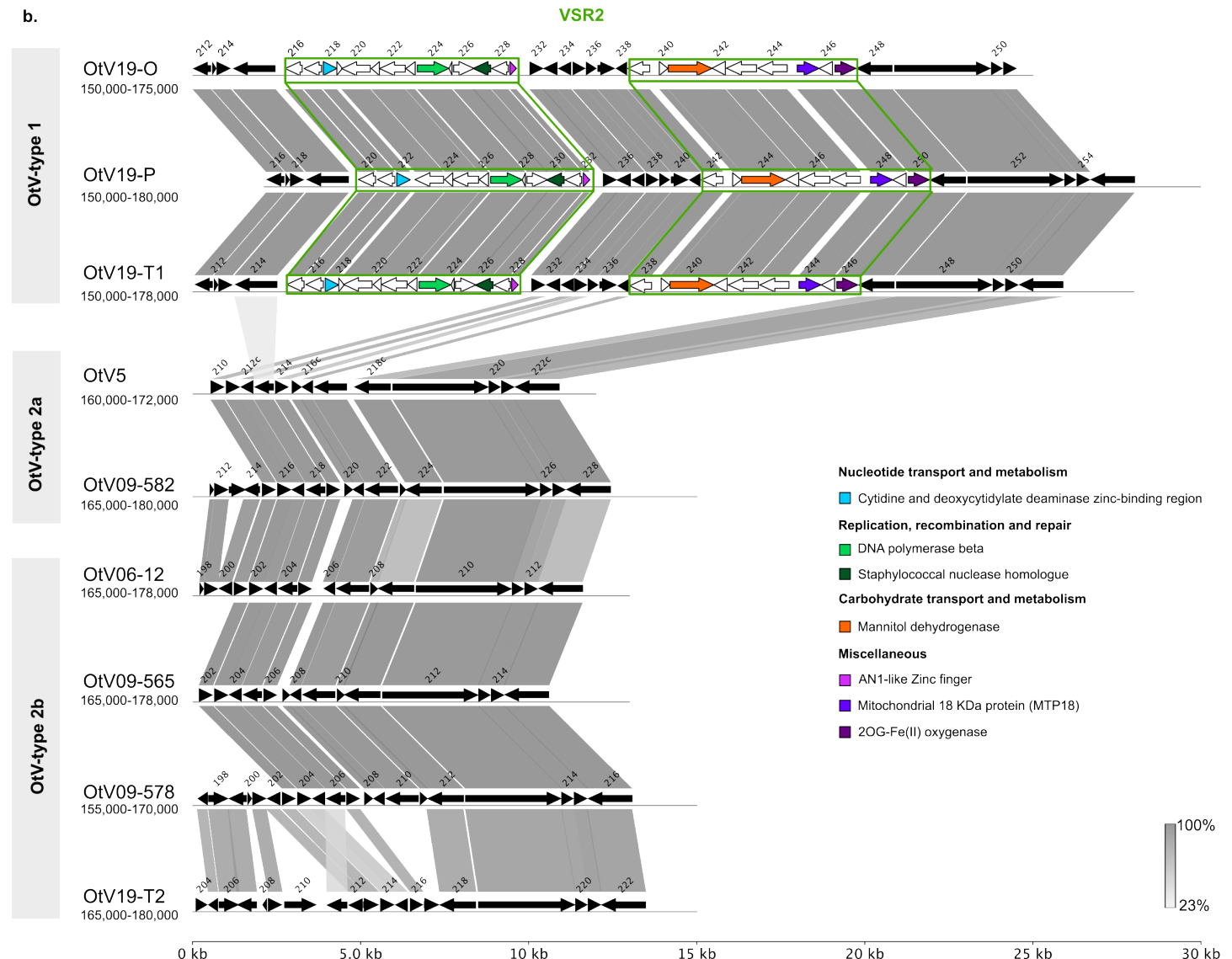

Supplement: supplementary-material [file supplementary-material.zip › FigureS6_veaf096.pdf]

Mitochondrial 18 kDa protein

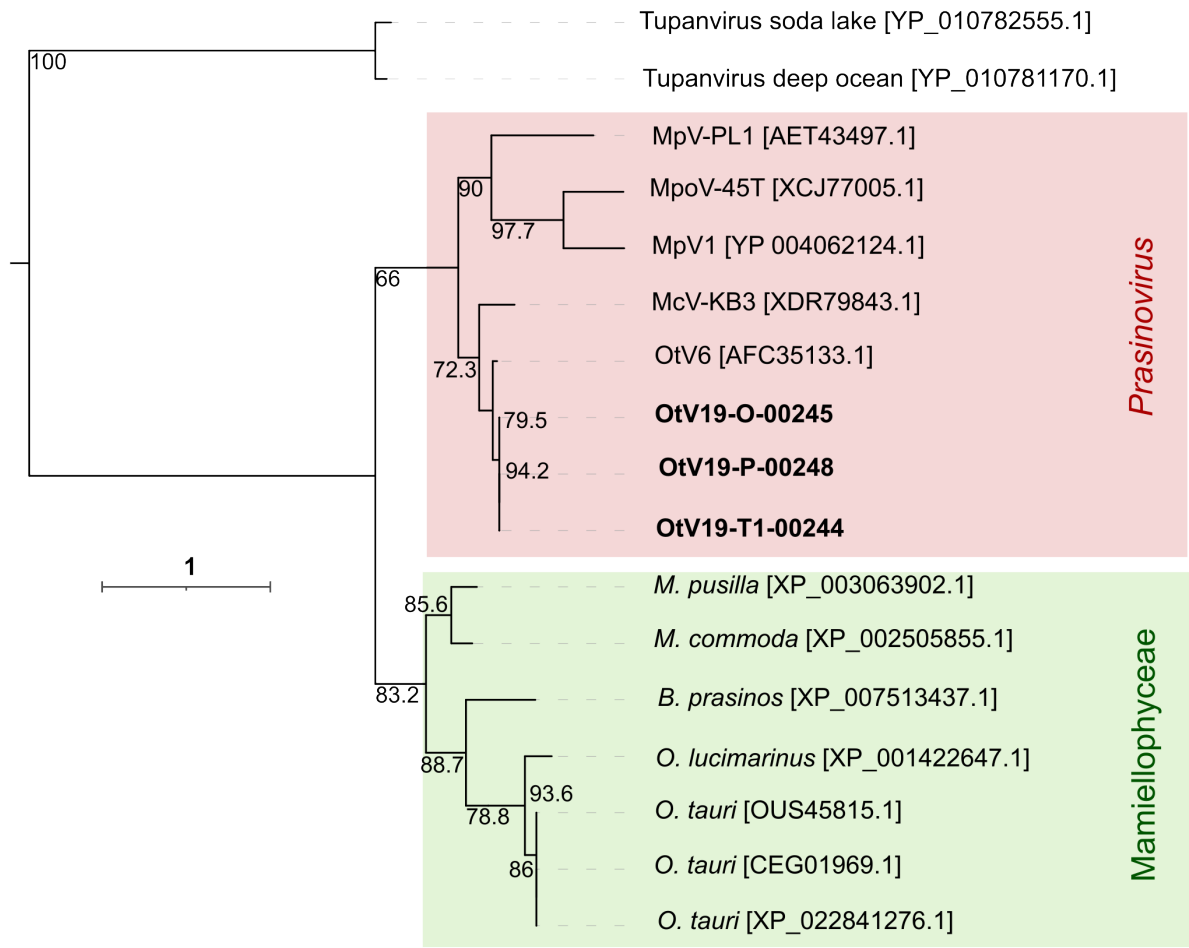

Supplement: supplementary-material [file supplementary-material.zip › FigureS7_veaf096.pdf]

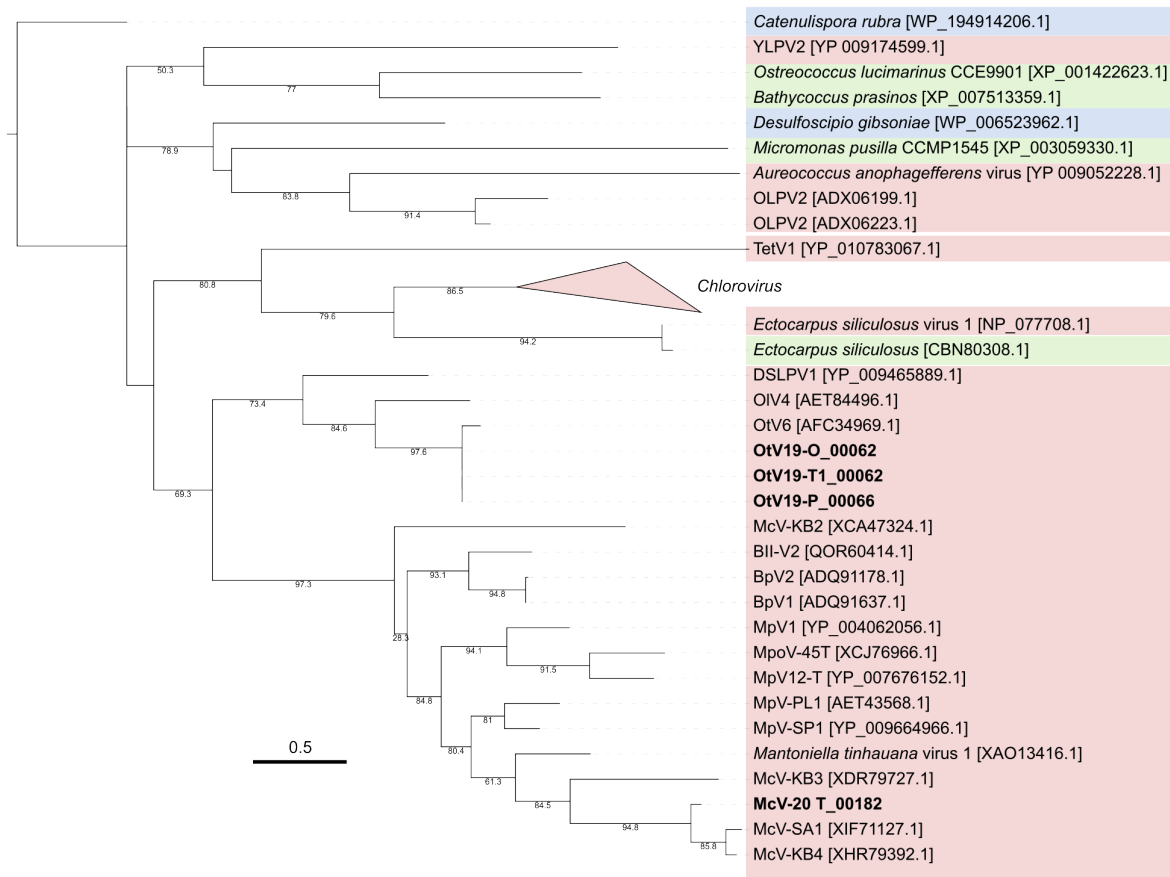

Supplement: supplementary-material [file supplementary-material.zip › FigureS8_veaf096.pdf]

**a. *O. tauri* RCC1108**

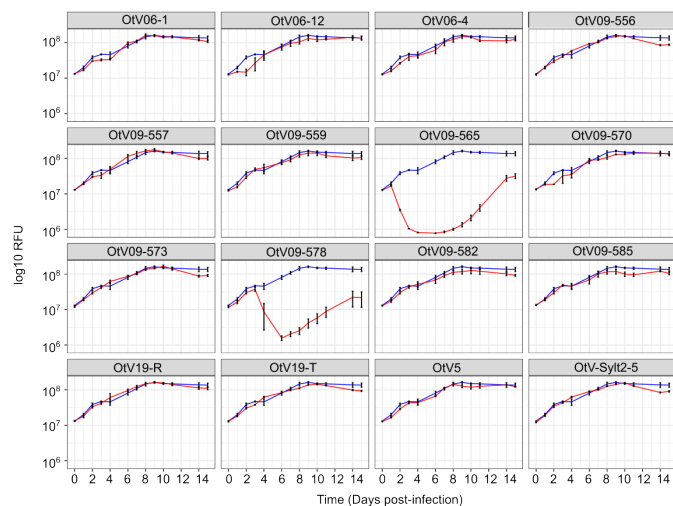

**b. *O. tauri* RCC1116**

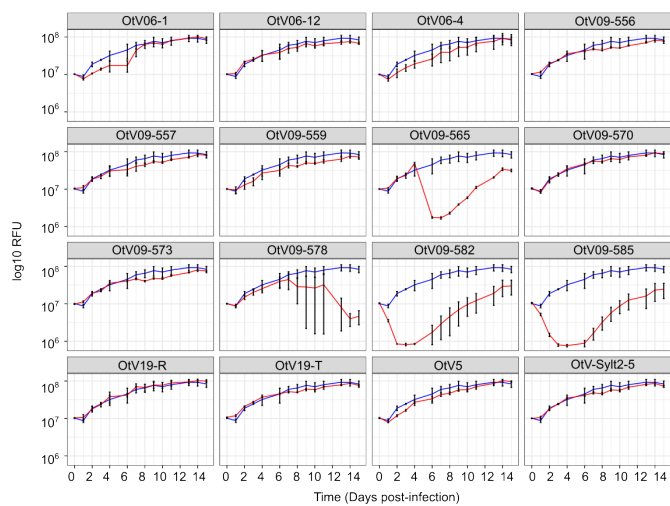

**c. *O. tauri* RCC1123**

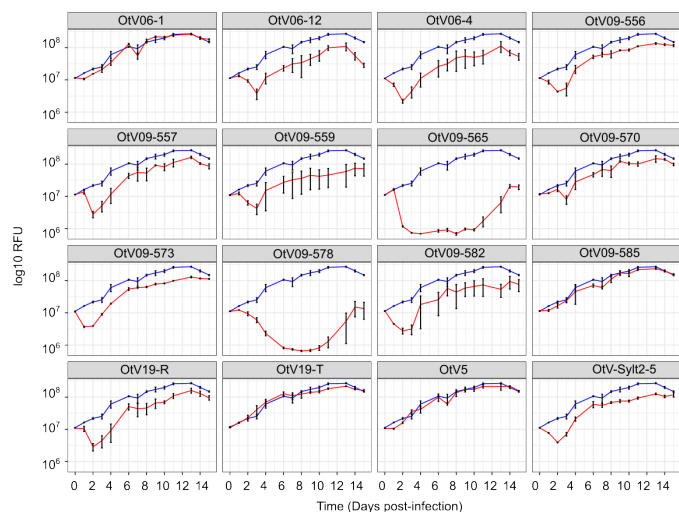

**d. *O. tauri* RCC4221**

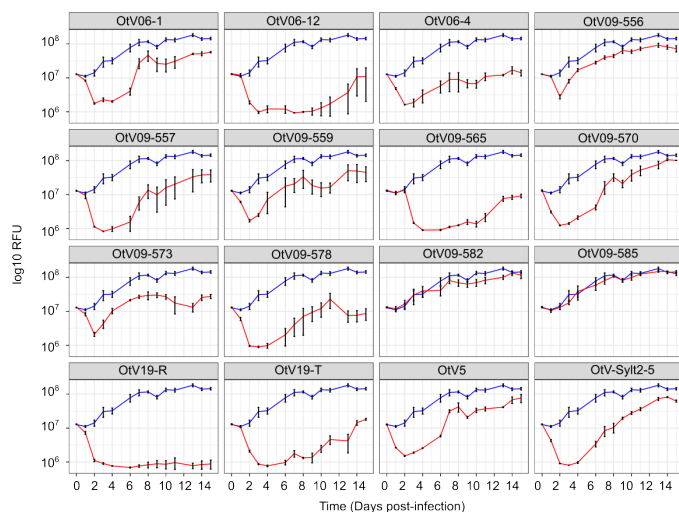

Supplement: supplementary-material [file supplementary-material.zip › FigureS9_veaf096.pdf]
